# Increase of mitochondrial DNA content and transcripts in early bovine embryogenesis associated with upregulation of mtTFA and NRF1 transcription factors

**DOI:** 10.1186/1477-7827-3-65

**Published:** 2005-11-14

**Authors:** Pascale May-Panloup, Xavier Vignon, Marie-Françoise Chrétien, Yvan Heyman, Manoel Tamassia, Yves Malthièry, Pascal Reynier

**Affiliations:** 1 Biologie de la Reproduction, Labo FIV, Centre Hospitalier Universitaire d'Angers, 4 rue Larrey, F-49033 Angers, France; 2 Inserm, U694, F-49033 Angers, France; 3 INRA, Biologie du Développement et Reproduction, UMR 1198 INRA/ENVA, F-78352 Jouy en Josas cedex, France; 4 University of Illinois, Dept of Veterinary Clinical Medicine, 1008 West Hazelwood Dr. Urbana, IL 61802, USA; 5 Centre Hospitalier Universitaire d'Angers, Laboratoire de Biochimie et Biologie Moléculaire, 4 rue Larrey, F-49033 Angers, France

## Abstract

**Background:**

Recent work has shown that mitochondrial biogenesis and mitochondrial functions are critical determinants of embryonic development. However, the expression of the factors controlling mitochondrial biogenesis in early embryogenesis has received little attention so far.

**Methods:**

We used real-time quantitative PCR to quantify mitochondrial DNA (mtDNA) in bovine oocytes and in various stages of in vitro produced embryos. To investigate the molecular mechanisms responsible for the replication and the transcriptional activation of mtDNA, we quantified the mRNA corresponding to the mtDNA-encoded cytochrome oxidase 1 (COX1), and two nuclear-encoded factors, i.e. the Nuclear Respiratory Factor 1 (NRF1), and the nuclear-encoded Mitochondrial Transcription Factor A (mtTFA).

**Results:**

Unlike findings reported in mouse embryos, the mtDNA content was not constant during early bovine embryogenesis. We found a sharp, 60% decrease in mtDNA content between the 2-cell and the 4/8-cell stages. COX1 mRNA was constant until the morula stage after which it increased dramatically. mtTFA mRNA was undetectable in oocytes and remained so until the 8/16-cell stage; it began to appear only at the morula stage, suggesting de novo synthesis. In contrast, NRF1 mRNA was detectable in oocytes and the quantity remained constant until the morula stage.

**Conclusion:**

Our results revealed a reduction of mtDNA content in early bovine embryos suggesting an active process of mitochondrial DNA degradation. In addition, de novo mtTFA expression associated with mitochondrial biogenesis activation and high levels of NRF1 mRNA from the oocyte stage onwards argue for the essential function of these factors during the first steps of bovine embryogenesis.

## Background

Mitochondria, which are maternally inherited organelles, perform several cellular functions, e.g. energetic metabolism, calcium and iron homeostasis, signal transduction, and apoptosis, and play a role in metabolic pathways such as those involved in the biosynthesis of heme, lipids, amino acids and nucleotides [1]. These mitochondrial functions are therefore likely to be critical determinants of early embryonic development at various levels including spindle organization, chromosomal segregation, cell-cycle regulation, and morpho-dynamic processes such as compaction, cavitation and blastocyst hatching [2].

Pre-existing oocyte components are critical during the interval between fertilization and the so-called maternal-embryonic transition (MET) when the transcriptional activity of the embryonic genome becomes fully functional. During this period the development of the embryo is supported by maternal RNAs, proteins and organelles stored in the ooplasm. The transcription of the embryonic genome start at the 2-cell stage in the cow, defining a step called minor activation of the embryonic genome [3]. During the first cell divisions there is a balance between maternal and embryonic transcripts. Indeed, embryonic transcription and the degradation of maternal mRNA are gradual processes [4]. When the embryo reaches the 8/16-cell stage, the MET occurs, marking the major activation of the embryonic transcription and explaining the sharp increase in the RNA level at the blastocyst stage [3]. Throughout the preimplantation period the gene expression pattern is not constant but varies according to the gene considered.

The active transcription of the mitochondrial genome starts at different developmental stages depending on the species. In mice, the mtDNA transcription occurs in the late 2-cell stage, whereas it occurs in the 4/8-cell stage in humans and in the 8/16-cell stage in cattle [5,6]. The molecular mechanisms responsible for this transcriptional activation of mtDNA during early embryogenesis are not well understood. Ubiquitous transcription factors, such as the nuclear respiratory factor 1 (NRF1) and the mitochondrial transcription factor A (mtTFA), are well known to regulate mtDNA transcription in various tissues. NRF1 transactivates the promoters of a number of mitochondrial-related genes including genes coding for respiratory chain subunits and mtTFA [7]. Mitochondrial TFA is a nuclear-encoded high-mobility group (HMG) box protein, which binds upstream of the light- and heavy-strand mtDNA promoters [8]. This transcription factor also regulates mtDNA replication, since the initiation of replication of the leading strand of mtDNA depends on an RNA primer produced by transcription from the light-strand promoter. Moreover, there is new evidence that mtTFA plays a role in the direct regulation of the mtDNA copy number [9]. A recent study on early mouse embryogenesis shows that sharp changes in the abundance of NRF1 and mtTFA mRNAs occur in the 8-cell stage, which is one cell cycle before changes appear in mitochondrial oxidative phosphorylation transcripts, although mtDNA replication does not occur until later in the development [10]. In contrast to mouse embryos, *in vitro *fertilized bovine embryos showed a significantly higher mtDNA copy number at the blastocyst stage [11]. Since the bovine blastocyst has a high mtDNA copy number, it offers a good mammalian model to study the regulation of the transcription of factors controlling mtDNA replication in the preceding stage.

The purpose of this study was to explore the variation of mtDNA and mitochondrial RNA (mtRNA) content through the different stages of early bovine embryogenesis and to investigate the possible role of NRF1 and mtTFA in the activation of mtDNA replication and transcription. To achieve this, we used the real-time polymerase chain reaction (PCR) to quantify mtDNA, and real-time reverse-transcription PCR (RT-PCR) to quantify mtDNA transcripts as well as NRF1 and mtTFA transcripts in metaphase II bovine oocytes and in bovine embryos at early stages of development.

## Methods

### In vitro production of bovine oocytes and embryos

Cumulus oocyte complexes (COCs) were obtained from bovine ovaries collected immediately after slaughter and transported to the laboratory in container maintained at 30°C. The content of antral follicles 2–8 mm in diameter was aspirated and recovered in a conical 50-ml tube containing 10 ml of HEPES-buffered M199 medium at 39°C. Oocytes were selected on the basis of their morphology and rinsed before *in vitro *maturation. COCs containing degenerated oocytes, oocytes with irregular ooplasm, and COCs with abnormal or expanded cumulus investments were discarded.

The maturation of the COCs was carried out as described in the literature [12]. Briefly, the COCs were matured *in vitro *for 22–24 h at 39°C under a humidified atmosphere of 5% CO_2 _and air in M199 medium supplemented with 10% fetal calf serum (FCS) (Life Technologies, Cergy, France), 10 μg/ml FSH (Stimufol, Mérial, Lyon France), 1 μg/ml LH and 1 μg/ml estradiol 17β (Sigma). At the end of the maturation period, cumulus-expanded oocytes where either inseminated *in vitro *with frozen-thawed semen, or dechoronized and retained if they present their first polar body (group of metaphase II oocytes before insemination). One ejaculate from a single bull was used throughout all the experiments. Eighteen hours after fertilization, presumptive zygotes had their cumulus cells removed by vortexing and were transferred into 50 μl microdrops of B2 medium (CCD-laboratories, Paris, France) supplemented with 2.5% of FCS and containing a layer of Vero cells for coculture according to a technique described elsewhere [12]. A total number of 610 in vitro matured oocytes were inseminated through 5 replicate experiments. Inseminated oocytes were distributed by groups of about 30 presumptive zygotes in microdrops. Oocytes that failed to divide at 28 hpi were collected at that time to constitute the group of uncleaved oocytes. Each microdrop was then allocated to the collection of one of the following specific developmental stage : 2-cell at 28 hpi, 4/8-cell at 48 hpi, 8/16-cell at 72 hpi, morula at 120 hpi and blastocyst at 168 hpi. Only the more advanced embryos in development for each stage, and morulas and blastocysts of highest visual quality were included in the study. Each sample was constituted by a single oocyte or embryo rinsed in 50 μl of PBS – immediately frozen in liquid nitrogen and individually stored at -80°C until assay.

DNA extraction and mtDNA quantification was performed on 105 oocytes and embryos: 15 single metaphase II oocytes collected just before insemination, 15 metaphase II oocytes, which had failed to cleave, and 15 isolated embryos at each of the developmental stages. RNA extraction and RNA transcript quantification were carried out on a similar series of 105 oocytes and embryos at the same developmental stage.

### DNA extraction

DNA was extracted from each single oocyte or embryo by means of the High Pure PCR Template Preparation Kit (Roche Diagnostics, Mannheim, Germany) according to the manufacturer's recommendations. The DNA was bound specifically to glass fibers following the combined action of a chaotropic agent (guanidine), a detergent (Triton X-100) and the enzyme proteinase K. After washing, the silica-bound DNA was eluted with 200 μl of pre-warmed (72°C) elution buffer and maintained at 4°C. The extraction efficiency, assessed as described elsewhere [13], was greater than 90%.

### RNA extraction and Reverse transcription (RT)

Poly(A) RNA was prepared from isolated single oocytes and embryos using the High Pure Viral RNA Kit (Roche Diagnostics, Manheim, Germany) following the manufacturer's instructions. Briefly, lysis was accomplished by incubation of the sample in a special Binding Buffer (4.5 M guanidine-HCl, 50 mM Tris-HCl, 30% Triton^® ^X-100) supplemented with poly(A) carrier RNA. The nucleic acids then bound specifically to the surface of glass fibers in the presence of a chaotropic salt. After washing, the silica-bound RNA was eluted with 50 μl of elution buffer and stored at -80°C until use. To confirm the absence of contaminating DNA, each RNA extract was subjected to the amplification protocol with the COX1 primer (see below) before reverse transcription.

Ten microlitres of each resultant poly(A) mRNA sample were used in duplicate. The RT-PCR reaction was carried out with the Advantage RT for PCR Kit (Becton Dickinson, Franklin Lakes, NJ, USA) following the manufacturer's instructions using a random hexamer mix to prime the RT reaction and to produce cDNA. Tubes were heated to 70°C for 2 mn to denature the secondary RNA structure and the RT mix was completed with 200 U of the MMLV RT enzyme. They were then incubated at 42°C for 1 hour to promote the reverse transcription of RNA, followed by incubation at 94°C for 1 mn to denature the RT enzyme. Each sample was completed to 50 μl with RNAse-free sterile water and stored at -80°C until use.

### Primer design

For mtDNA quantification, we used a couple of primers located in the COX1 gene (Table 1). The PCR product was a 190-bp DNA fragment. mtRNA quantification was performed using the same couple of primers. Because bovine sequences for mtTFA and NRF1 are currently unknown, we first used primers located in sequences highly conserved between species in order to amplify bovine sequences. The conserved sequences of NRF1 and mtTFA mRNAs were evaluated by nucleotide multiple-sequence alignments of several orthologues using Clustal W 1.83 [14]. Alignments of complete NRF1 coding sequences were performed using sequences available in RefSeq from Homo sapiens (NM_005011), Mus musculus (NM_010938) and Danio rerio (NM_131680). Alignments of complete mtTFA coding sequences were performed using sequences available in RefSeq from Homo sapiens (NM_003201), Mus musculus (NM_010938), and Rattus norvegicus (NM_131680).

**Table 1:** Primer couples and PCR conditions

**Gene**	**Primer sequence D**	**Primer sequence R**	**PCR**^1^	**AT**^2^	**CN**^3^
COX1	5'-AAA-TAA-TAT-AAG-CTT-CTG-ACT-CC-3'	5'-TCC-TAA-AAT-TGA-GGA-AAC-TCC-3'	190	56	4.8
mtTFA	5'-CAA-ATG-ATG-GAA-GTT-GGA-CG-3'	5'-AGC-TTC-CGG-TAT-TGA-GAC-C-3'	148	58	6.1
NRF1	5'-CCC-AAA-CTG-AGC-ACA-TGG-C-3'	5'-GTT-AAG-TAT-GTC-TGA-ATC-GTC-3'	162	58	5.6

^1 ^PCR: PCR product length/pb

^2 ^AT: Annealing temperature/degree C°

^3 ^CN: Copy Number in 1 ng of PCR product × 10^9^

After purification (High Pure Purification Kit, Roche Diagnostics, Mannheim, Germany) and sequencing of the PCR products, we designed bovine-specific primer couples. The PCR products were a 148-bp DNA fragment for mtTFA and a 162-bp DNA fragment for NRF1 (Table 1).

We also tried to quantify the housekeeping genes β-actin and histone H2A using primers described in the literature [15,16].

### Preparation of external standards

For each gene studied, PCR reactions were carried out under standard conditions with 100 ng of total bovine DNA, extracted from a piece of bovine muscle, in a 50 μl volume: 1.5 mM MgCl_2_, 75 mM Tris-HCl (pH 9 at 25°C), 20 mM (NH_4_)_2_SO_4_, 0.01% Tween 20, 50 pmol of each primer, 200 μM of each dNTP and 2 units of GoldStar DNA polymerase (Eurogentec, Belgium). Each of the 30 cycles consisted of a denaturation step of 30 seconds at 94°C, a hybridization step of 30 seconds at 58°C, and an extension step of 1 min at 72°C. The PCR products were purified using the High Pure Purification Kit (Roche Diagnostics, Mannheim, Germany) and quantified by spectrophotometry. The quality of purification was checked by means of the 260/280 ratios, values between 1.8 and 2.0 being considered acceptable. It was assumed that 1 ng of a 100 bp product contained 9.1 × 10^9 ^molecules of double-stranded DNA. Table 1 shows the number of molecules of double-stranded DNA per nanogram of each of the PCR products obtained. Several serial dilutions were then made in order to assess the concentrations of a known number of templates. These were used as external standards for real-time PCR. The serial dilutions were all stored at -20°C in single-use aliquots.

### Quantification of mtDNA and cDNA

We used a Roche LightCycler to determine the mtDNA and the cDNA copy number using the LightCycler FastStart DNA master SYBR Green 1 kit (Roche, Mannheim, Germany) as described elsewhere [13]. Briefly, 20-μl PCR reaction mixtures were prepared as follows: 1× buffer containing 4 mM MgCl_2_, 0.2 mM dNTPs, 0.5 μM of both primers for each gene, SYBR Green I dye, 0.25 U HotStart Taq DNA polymerase and 10 μl of the extracted mtDNA or 10 μl of the cDNA obtained or 10 μl of standard with a known copy number. The reactions were performed as follows: initial denaturing at 95°C for 7 min and 40 cycles at 95°C for 1 s, 56–59°C for 5 s, and 72°C for 13 s. The SYBR Green fluorescence was read at the end of each extension step (72°C). A melting curve (loss of fluorescence at a given temperature between 66°C and 94°C) was analyzed in order to check the specificity of the PCR product. For each run, a standard curve (log of the initial template copy number on the abscissas, and the cycle number at the crossing point on the ordinates) was plotted using five 10-fold serial-dilutions (100–1,000,000 copies) of the external standard. This standard curve, which depends on the efficiency of the PCR reaction, allowed the determination of the starting copy number of mtDNA or of the cDNAs in each sample. All samples were tested twice. The raw data was then multiplied by 20 to calculate the total mtDNA content in each oocyte or embryo. For the transcripts studied, we multiplied the raw data by 25 to express the cDNA level for each oocyte or embryo treated. The precision of the real-time PCR quantification was assessed as described elsewhere [13]. The CV of the intra-assay and inter-assay values ranged from 3.9% to 9.1% and from 9.3% to 12.7% respectively.

### Statistical analysis

Since the distribution of the variables analyzed was non-Gaussian, all comparisons were made using the non-parametric Mann-Whitney and Kruskal-Wallis *U*-tests. Results are given as mean values ± SE. Statistical analysis was performed with SPSS software, version 10.1 (SPSS, Chicago, IL, USA) and differences were considered significant at *p *< 0.05.

## Results

In this study, the in vitro cleavage rate was 88% (as assessed by the number of embryos with 2 cells or more at 48 hpi) and 54,4% of cleaved oocytes developed to the blastocyst stage at day 7.

### mtDNA copy number

**Figure 1:**
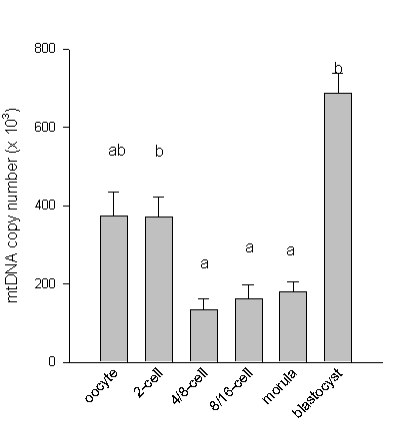
Comparison of mtDNA content in bovine oocytes and embryos at various stages of development. The mtDNA content decreases between the 2-cell and the 4/8-cell stages (*p *= 0.0008); in contrast, it increases sharply between the morula and the blastocyst stages (*p *< 0.0001). Bars with different superscript differ significantly.

The mean mtDNA copy number at each embryonic stage is shown in Figure 1. There was no statistical difference between the mean mtDNA copy number in metaphase II oocytes (373,000 ± 63,000) and 2-cell embryos (371,000 ± 52,000). In contrast, the mtDNA content was significantly higher in 2-cell embryos compared to 4/8-cell embryos (p = 0.0008). There was no significant variation of the mean mtDNA copy number between the 4/8-cell stage (135,000 ± 28,000), the 8/16-cell stage (163,000 ± 36,000) and the morula stage (180,000 ± 26,000). However, there was a considerable increase in the mtDNA copy number at the blastocyst stage (688,000 ± 50,000) (p < 0.0001).

### mRNAs quantification

It seems illusory to seek a detectable and constant housekeeping gene during early embryogenesis. Indeed, this is a much debated subject [10,15-17]. In our study β-actin levels remained low or undetectable during the first stages (oocyte, 2-cell, 4/8-cell, 8/16-cell stages), and then increased dramatically during the morula stage. Moreover, histone H2A was not detectable until the morula stage (data not shown). The reproducibility of the results (two RT-PCRs for each sample tested twice for each gene) and the homogeneity at a given embryonic stage led us to express our results in arbitrary units per oocyte or embryo.

**Figure 2:**
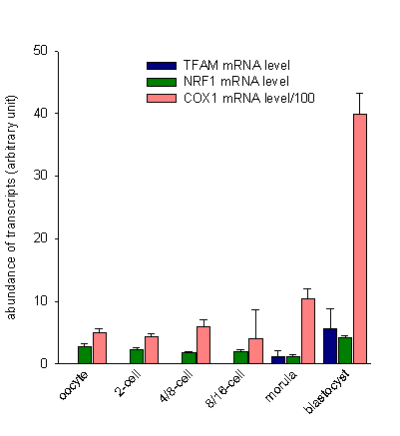
Comparison of COX1, mtTFA and NRF1 transcript levels in oocytes and at various stages of bovine embryonic development. Levels of COX1 mRNA remain constant from the oocyte to the 8/16-cell stage, and then increase sharply from the morula stage onwards (*p *= 0.002). mtTFA mRNA was not detected before the morula stage. The abundance of this transcript increased dramatically at the blastocyst stage (*p *< 0.0001). The abundance of NRF1 transcripts remained practically constant from the oocyte to the morula stage, after which it increased significantly up to the blastocyst stage (*p *< 0.0001).

The levels of mitochondrial COX1 mRNA, which remained roughly constant from the oocyte to the 8/16-cell stage, increased sharply after the morula stage (p = 0.002) (Figure 2). The mtTFA mRNA was undetectable until it appeared at the morula stage. The quantity of this transcript increased dramatically at the blastocyst stage (p < 0.0001) (Figure 2). The quantity of NRF1 transcripts remained practically constant from the oocyte to the morula stage, after which it increased significantly up to the blastocyst stage (p < 0.0001) (Figure 2).

### Uncleaved oocyte

**Figure 3:**
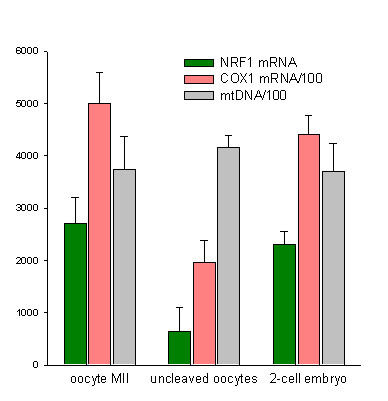
Comparison between uncleaved oocytes and 2-cell embryos (both collected at 28 hpi), and oocytes collected before insemination. Although, the mean mtDNA copy numbers did not differ between these three groups, COX1 and NRF1 transcripts were significantly fewer in uncleaved oocytes (*p *< 0.0001).

There was no significant difference between mean mtDNA copy number of post insemination uncleaved oocytes (415,000 ± 24,000) and 2-cell embryos (371,000 ± 52,000) both collected 28 hpi. However, the number of COX1 and NRF1 RNA transcripts was significantly lower in the uncleaved oocytes as compared to the embryos that cleaved or to the oocyte before insemination (p < 0.0001) (Figure 3). Moreover, the mean mtDNA copy number and transcripts levels are similar in oocytes collected before insemination and 2-cell embryos (Figure 3).

## Discussion

The mean mtDNA copy number per bovine metaphase II oocyte reported here (373,000) is comparable to the 260,000 copies/oocyte first determined in 1982 [18] using the hybridization technique, and very close to the 377,000 copies/oocyte recently found by our group using real-time quantitative PCR [19]. Experiments on bovine oocytes have shown that the mtDNA content, in at least some species, is related to the competence of development to the blastocyst stage [19]. The progression of the mtDNA content during early embryogenesis *in vitro *has been performed only in mouse eggs [20]. This study, performed on pooled oocytes and embryos with the Southern-blot technique, showed that the mtDNA content remained constant from the oocyte to the implantation stage. These results have led to the general belief that mtDNA replication does not occur until after implantation [21]. This result has just been confirmed by quantitative PCR analysis on mouse embryos [10]. However, the fact that the mtDNA copy number remains stable during early mouse embryogenesis could be due to a balance between the degradation and synthesis of mtDNA. Indeed, a recent report has indicated that mtDNA replication occurs in pronuclear and 2-cell stage mouse embryos [22]. In contrast to the mouse model, a marked increase of mtDNA replication from the blastocyst stage onwards was found in bovine embryos. Thus, DNA replication is disconnected from the implantation event (21 days in the bovine), and occurs at an earlier embryonic stage in this species. We observed a significant reduction of about 60% in the mtDNA content between the 2-cell and the 4/8-cell stages. This finding is reinforced by the fact that the metaphase II oocytes and the 2-cell embryos had similar high mtDNA levels, whereas the 4/8-cell, the 8/16-cell embryos and the morulas had similar low mtDNA levels (Figure 2). This drastic reduction of mtDNA content argues in favor of active destruction rather than a reduced turnover of mtDNA molecules. It has been demonstrated that in the course of mammalian embryogenesis, the paternal mtDNA is destroyed at the same stage by a mechanism involving the proteasome [23]. The active destruction of mtDNA would be compatible with the bottleneck hypothesis proposed to explain the homogeneity of the transmitted mitochondrial genomes. This phenomenon of restriction-amplification in the mtDNA copy number seems to occur in multiple steps during oogenesis and embryogenesis [24].

COX1 is a respiratory chain protein encoded by mtDNA. We found that COX1 mRNA increases sharply from the morula stage onwards. The same pattern of expression has been described for cytochrome b mRNA, which is another respiratory chain transcript encoded by the mitochondrial genome [25]. According to several reports [5,6], the onset of mitochondrial transcriptional activity appears to occur at the same time as the MET. Before this, during the early stages of bovine embryogenesis, the level of mitochondrial transcripts remains roughly constant. This observation is supported by other studies in which the inhibition of mitochondrial transcription permitted embryonic development in the mouse up to the blastocyst stage [8,26]

In mammals, mtTFA has been isolated only in humans [27], in mice [28], and in rats [29]. The mouse mtTFA gene, estimated to span about 10 kb, consists of 7 exons and 6 introns [30]. The NRF1 gene sequence in mice, sheep, fish and humans is partially or totally known. The human NRF1 gene spans 65 Kb and comprises 11 exons and 10 introns [31]. Since the sequences of bovine mtTFA and NRF1 genes remain unknown, we selected nucleotide sequences of primer couples among the exon sequences that are highly conserved between species to obtain bovine PCR products. We retained only the primer couples that yielded single, pure PCR products. Upon sequencing, these PCR products showed a homology of 76% with human mtTFA and a homology of 89% with human NRF1.

NRF1 and mtTFA are ubiquitous factors well known to regulate mtDNA transcription and replication in various tissues. The critical role of mtTFA in embryogenesis has been demonstrated in transgenic experiments. Indeed, in knockout mice the implication of mtTFA in the regulation of the mtDNA copy number has been demonstrated together with its essential involvement in mitochondrial biogenesis and embryonic development [8]. In our study, we found no mtTFA expression before the morula stage. The appearance of mtTFA transcripts is concomitant with the increase of mitochondrial mRNA and just precedes the increase of the mtDNA copy number. This result strongly suggests that the activation of mitochondrial biogenesis in the bovine embryo occurs between the 8/16-cell and the morula stages under the impulse of mtTFA.

Moreover, it has been shown that the homozygous disruption of the mouse NRF1 gene leads to embryonic death around the time of implantation. The depletion of mtDNA occurring between fertilization and the blastocyst stage suggests that NRF1 is required for mitochondrial maintenance *in vivo*. In this mouse model, the transcription of NRF1 occurred not only during oogenesis but also in early embryogenesis [32]. We found that NRF1 expression was constant up to the blastocyst stage. Thus, it is likely that NRF1 mRNA pre-exists in the oocyte and that a balance is established between the degradation of maternal transcripts and the synthesis of embryonic mRNA. The expression of NRF1 all through early embryogenesis may be necessary to maintain mitochondrial activity and other vital embryonic functions without the intervention of mtTFA [33]. This hypothesis is supported by the finding that homozygous NRF1 knockout embryos died significantly earlier than homozygous mtTFA knockout mouse embryos (at an average age of 6.5 days *versus *10.5 days). Conversely, the onset of mitochondrial biogenesis at the MET stage under the dependence of mtTFA may be initiated either by NRF1, progressively unmasked to become functional, or by other transcription factors known, at least in humans, to act on the mtTFA promoter [34].

We found that bovine oocytes that failed to cleave at 28 hpi contained significantly fewer transcripts implicated in mitochondrial biogenesis (COX1 and NRF1 mRNAs) than 2-cell stage embryos (collected at the same time) as well as potentially fertilizable oocytes collected before insemination. This finding substantiates the hypothesis that mitochondrial quality is closely related to the fertilizability of the oocyte and to the developmental capacity of the embryo [35]. Indeed, in the case of human oocytes, the developmental potential of the embryo has been shown to be related to the ATP content of the cells [36]. Furthermore, the injection of a small number of mitochondria into mouse oocytes prevents these cells from undergoing apoptosis [37]. However, further investigation will be needed to establish whether the impairment of the factors of mitochondrial biogenesis is the central cause of fertilization failure or merely incidental to a vaster death process.

To our knowledge, this is the first study on a bovine model and using isolated oocytes and embryos. We have determined the kinetics of mtDNA replication and transcription during early bovine embryogenesis in vitro and studied the expression of mtTFA and NRF1, the two main regulators of mitochondrial biogenesis. Our results support the hypothesis that these factors play a critical role in mitochondrial biogenesis during early embryogenesis.

## Authors' contributions

MPP carried out the DNA and RNA extraction, the PCR (and the RT-PCR) reactions and drafted the manuscript. VX and HY participated in collecting the oocytes and embryos and collaborated in the design and the coordination of this study. CMF and TM have made substantial contributions to the analysis and interpretation of the data. They have been involved in revising the manuscript critically for its content. MY has been involved in revising the manuscript critically for his content. RP conceived the study and participated in its design and coordination and helped to draft the manuscript. All the authors have read and approve of the final manuscript.
